# The use of CRISPR to generate a whole-gene humanized *MAPT* and the examination of P301L and G272V clinical variants, along with the creation of deletion null alleles of *ptl-1, pgrn-1* and *alfa-1* loci

**DOI:** 10.17912/micropub.biology.000615

**Published:** 2022-09-19

**Authors:** Jeremy Lins, Christopher E. Hopkins, Trisha Brock, Anne C. Hart

**Affiliations:** 1 Department of Neuroscience, Brown University, Providence, RI 02912; 2 InVivo Biosystems, Eugene, OR, 79402; 3 Robert J. & Nancy D. Carney Institute for Brain Science, Brown University, Providence RI 02912

## Abstract

To study important genes involved in Frontotemporal Dementia ( *MAPT* , *GRN* and *C9orf72* ), we created deletion alleles in the three orthologous genes ( *ptl-1* , *pgrn-1* , and *alfa-1* ). Simultaneously, we replaced the *C. elegans* *ptl-1* gene with the predicted orthologous human *MAPT* gene, often called whole-gene humanization, which allows direct assessment of conserved gene function, as well as the opportunity to examine consequences of clinical disease-associated patient variations. Each gene was manipulated using a different selection strategy, including a novel strategy using an *unc-18* mutation rescue technique. Clinical *MAPT* ALS/FTD missense variants G272V and P301L were successfully inserted in *hMAPT* . Neither *ptl-1* loss or clinical variants caused neuronal defects in young adult or aged *C. elegans* , based on examination of glutamatergic phasmid neurons. Yet, we noted decreased survival to day 9 in the P301L *hMAPT* strain, compared to control strains. Based on these results, we comment on strategies for humanization, including the importance of confirming *C. elegans* gene predictions and identifying loss of function defects for each gene before embarking on humanization, and we report the creation of strains and a new gene-editing selection strategy that will be useful for future studies.

**Figure 1. Creation of deletion alleles and humanization with their functional consequences.**
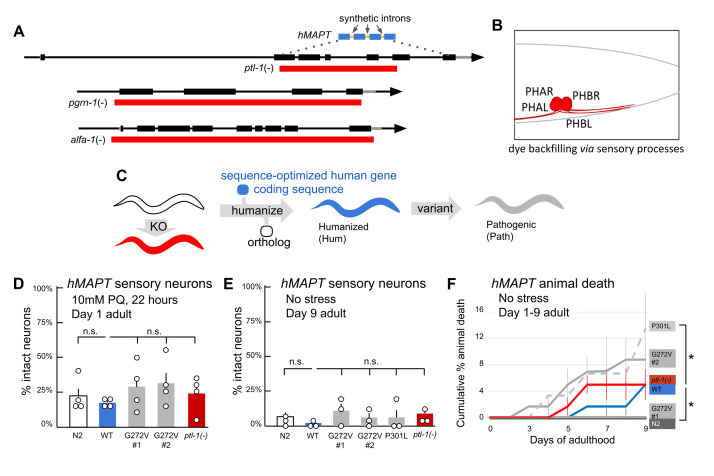
Figure legend. (A) Strategy for deletion of *ptl-1* , *pgrn-1* and *alfa-1* , including humanization of *ptl-1* by inserting MAPT *.* Native genome is shown in black, exons are black boxes, deleted regions shown in red and humanization shown in blue, synthetic introns indicated with arrows. (B) Dye filling assay. Four glutamatergic neurons in the tail can backfill with fluorescent lipophilic dyes *via* sensory endings that are normally exposed to the environment: PHA and PHB, left and right. Process degeneration or neuron loss is detected as a lack of dye backfilling in the cell body. Pre-exposure to paraquat induces dose-dependent neurodegeneration and can reveal latent hypersensitivity to stress. Live animals on cultures dishes were scored here and loss of both PHA and PHB on one side of the animal was unambiguous; loss of one neuron was difficult to detect. Therefore, degeneration reported here as % intact is likely an underestimate of neurons lost. (C) Schematic of *ptl-1* humanization strategy. Initially, a knockout (KO) of *ptl-1* is made. Then, *C. elegans ptl-1* coding exons and introns are replaced with coding sequence for the human gene and artificial introns. Finally, variants are introduced into the human *MAPT* sequence. The phenotypic consequences of these manipulations are analyzed. (D) Percent intact Day 1 adult animals based on dye backfilling of phasmid neurons after 22 hours on 10mM paraquat. Each data point is one of four independent trials reported with 20 animals each trial/genotype (except only 10 animals in one of the G272V trials). SEM and mean for each trial indicated. Student’s T-test, two-tailed for disease variants *versus* *hMAPT* wild type (WT) animals. N2 are standard *C. elegans* containing an unedited *ptl-1* allele. WT animals are *hMAPT* and presumably express *MAPT* as shown in B. (E) Percent intact Day 9 adult animals, assayed as in D, but no paraquat pre-treatment and 15 animals each trial/genotype. SEM and mean for each trial indicated. Statistical analysis and genotypes as in D. (F) Cumulative percentage of animals that died when aging animals to 9 days of adulthood. *hMAPT* *P301L* shows increased death at Day 9, compared to *hMAPT* *WT* (p<0.005) *; hMAPT WT* shows increased death versus N2 (p<0.005). Each of the three trials started with 20 animals per genotype.

## Description

Here, we focus on *C. elegans* orthologs of three human genes with clinical variants that lead to Frontotemporal Dementia (FTD), with associated neurodegeneration and inevitable death in patients (Alberici et al., 2004; Bronner et al., 2005). As a resource for the community, we created deletion knockout (KO) alleles for *C. elegans* *MAPT* ortholog *ptl-1* , for the *GRN* ortholog *pgrn-1* (Hsiung & Feldman, 2007), and for *C. elegans C9orf72* ortholog *alfa-1* (Gossye et al., 2015) (Panel A). To create a *ptl-1* KO, we CRISPR-engineered a deletion starting within the second exon and removing sequences through the beginning of the sixth exon; this removes the conserved protein domains that are shared by all *ptl-1* isoforms (Panel A). The *pgrn-1* and *alfa-1* KOs both remove all exons and introns, but leave most of the non-coding exons and flanking sequences intact. Complete loss of function alleles, *ptl-1(tgx30)* , *pgrn-1(tgx81)* , or *alfa-1(tgx53)* , here referred to as *ptl-1(-)* , *pgrn-1(-)* , and *alfa-1(-)* , respectively, did not cause lethality, which indicates these genes are not essential for viability in *C. elegans* .

The *pgrn-1* KO used a novel *unc-18* co-conversion rescue strategy (see Methods). This new co-CRISPR selection tool uses restoration of wild type phenotype from an *unc-18* loss of function insertion allele. The *unc-18* co-conversion approach is convenient because injections are performed in immotile *unc-18* animals and the co-conversion marker phenotype is restoration of wildtype activity. As a result, this provides to the community another co-CRISPR selection tool that may have advantages in certain contexts.

Confirming gene conservation often requires cross-species expression and experimental evidence confirming cross-species function. Frequently, studies of protein function, the consequences of clinical variants, and other preclinical studies can be efficiently carried out in “humanized” laboratory animal models. For *ptl-1,* we undertook such studies and created humanized *MAPT* ( *hMAPT* ) to examine patient variants. The construction of humanized *C. elegans* model and variant installation is shown in Panel C. An efficient strategy is genome editing to first remove most or all coding exons, as well as introns from the *C. elegans* gene (Panel C), creating an unambiguous complete loss of function allele. Then, sequences encoding the putative human ortholog are inserted creating a wild type (WT) humanized gene that can be tested for functional rescue. Note that because *ptl-1* loss of function had no phenotypic defects in our assays, it remains uncertain if the humanized *MAPT* gene is functional and is a verified genetic background for testing variants.

When human variants are inserted in the humanized gene, they may be benign, known deleterious alleles, or variants of unknown significance. Using CRISPR gene editing approaches, we inserted the human MAPT coding sequence as a gene replacement of the *ptl-1* locus from the second to last exon of the longest isoform. Next, variants were installed in the *hMAPT* locus using CRISPR gene editing approaches to create models of two clinical variants observed in patients with MAPT-associated pathologies, G272V and P301L (Panel C).

To assess phenotypic consequences, we examined the *hMAPT* strains for neurodegeneration using a dye backfilling assay (Perkins et al., 1986). If any of the four phasmid neurons in the tail are unable to take up dye from the environment, this indicates either neuron loss or defects in the sensory process that contacts the environment (Panel B). Pre-treatment with paraquat can reveal neuronal hypersensitivity to oxidative stress in this assay (Baskoylu et al., 2018). Older animals can also be examined to reveal age-dependent neurodegeneration (Faber et al. 1999). First, we examined *hMAPT* and *ptl-1(-)* young adults for oxidative stress-induced neurodegeneration. Increased neurodegeneration was not seen in either genotype (Panel D), however, high lethality was observed in N2 suggesting that in the future a shorter treatment of PQ or a lower concentration of PQ may reveal defects. We also did not detect increased neurodegeneration in aged animals (Panel E). We conclude that *ptl-1* loss of function does not cause paraquat-induced dye filling defects.

Next *,* we examined the phenotypic consequences of patient variants as these might be predicted to cause gain-of-function defects. For this analysis, we examined *hMAPT* *WT,* as well as *hMAPT* *P301L* and *hMAPT G272V.* We found that these *hMAPT* clinical variants do not show predicted gain of function neurodegenerative defects, in either young or old *C. elegans* . While aging animals for these studies, we also tracked survival from Day 1 through Day 9 of adulthood (Panel F). *hMAPT* *P301L* showed increased death at Day 9, compared to *hMAPT* *WT* (p<0.005) *. hMAPT WT* shows increased death versus N2 (p<0.005). We noted that *hMAPT G272V* showed inconsistent survival defects; this may be due to a background mutation despite backcrossing to wild type strains four times. We only have one *hMAPT* *P301L* allele; this shows decreased survival compared to *hMAPT WT* . But, to draw conclusions about a potential gain-of-function defect caused by *P301L,* another allele or additional studies will be required. Note that because KO *ptl-1(-)* animals do not have defects in neurodegeneration or the survival assays, any *P301L* defects are likely not due to *ptl-1* loss of function.

Based on this and other work, we believe these results highlight the importance of identifying *C. elegans* gene loss of function defects before humanizing and inserting variants when modeling loss of function disorders. When modeling gain of function disorders, a *C. elegans* loss of function phenotype may not be essential. Instead, independent lines of evidence will be required to validate any potential gain of function defects observed in the humanized disease allele. These might include comparison to benign variants, confirming accumulation of expected toxic proteins, ameliorating defects with established therapies, and/or confirming genetic or biochemical interactions already reported in the literature.

## Methods

Neurodegeneration was scored by ability to backfill with DiI (Molecular Probes). Animals were scored as intact if no neuron loss was detected, but note that at a magnification level of 12.5x with moving animals on culture dishes, loss of a single neuron will be missed in some animals. Therefore, animals with 3 or 4 neurons are challenging to distinguish and our results are likely an underestimate of affected neurons. Animals were scored blinded as to genotype. For Day 1 studies, L4 animals were picked to 10mM paraquat NGM plates spread with OP50 and scored the next day. For Day 9, L4 animals were serially passed to new plates (without FUDR) until the 9th day of adulthood. Then, dye filling was undertaken as above, but without paraquat.

Survival was examined while aging animals for neurodegeneration assessment at Day 9; survival was simultaneously assessed in the same population. Animals were censored if they bagged, burst, or escaped.

Knockout (KO) alleles were generated by deletion of the coding sequences and intervening exons using template-driven, CRISPR/Cas9-mediated homologous recombination. For each gene KO, a different co-conversion strategy was used: *pha-1* for the *ptl-1* KO (Ward, 2015) *dpy-10* for the *alfa-1* KO (Paix, et al 2017) and *unc-18* for the *pgrn-1* KO. Two guide RNAs (sgRNAs) were selected to flank coding sequences for each gene. We use two sgRNAs to avoid any issues with poor efficiency of a given sgRNAs. We find it more efficient to start with two sgRNAs; this avoids redesign and reinjection, if the first sgRNA has a low cutting efficiency. The donor template sequence was a single stranded oligodeoxynucleotide (ssODN) containing 35 base pair (bp) homology arms, a 3-frame stop sequence, and an XhoI restriction site. To aid in re-editing of the locus, the *pgrn-1* KO ssODN also contained a zebrafish sgRNA sequence which does not cut the *C. elegans* genome, but we know is a highly efficient sgRNA sequence for activating Cas9 targeted cutting. The sgRNAs, ssODNs, and Cas9 protein were injected into GE24 *(pha-1* co-conversion), N2 ( *dpy-10* co-conversion), or COP1403 ( *unc-18* co-conversion). Candidate edited lines were selected from the F1 population based on a visual detection of the co-conversion marker and screened by PCR for gene deletion. Homozygous deletion lines were confirmed by sequencing. Additionally, the co-conversion loci were validated as wild-type by sequencing.

*unc-18* co-conversion utilizes rescue of the *knu409* allele containing a 56bp insertion (CCTCCCGTTCGCCTGGGACATCTAAATAAATAAAGATGTCCCAGGCGAACGGGAGG) between amino acids 392 and 393 of the native *unc-18* gene. The 56bp insertion codes for an early stop and animals containing this allele (COP1403) have an uncoordinated movement phenotype. To use the co-conversion strategy, COP1403 animals are grown on HB101 bacteria for improved health (Frøkjær-Jensen et al., 2012) and injected with an sgRNA and ssODN (see Reagent Table Part 2) designed to correct the coding sequence back to wild-type. The sgRNA and ssODN for restoration of the *unc-18* locus and the sgRNA and ssODN for the target gene edit locus ( *pgrn-1* or *alfa-1* in this study) are included in the injection mix along with Cas9 protein. After injections, F1 animals that have rescued movement are isolated and screened by PCR for co-conversion at the locus of interest. The rate of co-conversion observed for the *pgrn-1* KO was 64%. PCR of the *unc-18* locus is recommended to ensure that the *unc-18* rescue is homozygous.

Humanization of *ptl-1* to create *hMAPT* was undertaken using a gene-swap method (McCormick et al., 2021). The majority of the coding sequence for the *C. elegans* MAPT ortholog *ptl-1* was replaced with an expression-optimized version of the 441 amino acid human MAPT ( *hMAPT* ) Tau-F/2N4R isoform (UniProt Identifier: P10636-8). To optimize the human coding sequence for *C. elegans* expression, we used the *C. elegans* codon adapter (Redemann et al., 2011) to select *C. elegans* preferred codons and introduced 3 synthetic introns. To preserve normal gene expression, the long intron between exons 1 and 2 was maintained and a portion of the coding sequence (exon 1 and some of exon 2) was fused to the introduced cargo of the human coding sequence. To create the gene insertion, one sgRNA targeted a sequence in the second exon of *ptl-1* and the other sgRNA targeted sequence after the final exon of the longest *ptl-1* isoform, isoform b. Matching these sgRNA sites, a donor homology plasmid was constructed using approximately 500bp homology arms, a P2A self-cleaving peptide, codon-optimized *hMAPT* 2N4R sequence, and a hygromycin-resistance selection cassette. The P2A self-cleaving peptide allows for the native PTL-1 coding sequence from introns 1 and 2 to be separated from the *hMAPT* coding sequence leaving only a residual proline on front of the *hMAPT* protein sequence after translation (Ahier & Jarriault, 2014). Injections were into N2 wild type animals of the donor homology plasmid, the two sgRNA plasmids, a plasmid expressing Cas9, and anti-array selection plasmids were performed (Frøkjær-Jensen et al., 2012). Positive selection used hygromycin B and arrays bearing lines were selected against by confirming lack of red fluorescence and *hs::peel-1* induced death. PCR was used to identify founders as *hMAPT* sequences integrated into the *C. elegans* genome and progeny were screened again to confirm that arrays were not present. Validation of insertion utilizes whole gene sequencing of the *hMAPT* integration locus to confirm construct contained transgene with desired sequence. Expression of the *hMAPT* was confirmed by isolating RNA, converting to cDNA, and PCR amplification of *hMAPT* from the cDNA.

Clinical variants were similarly inserted into *hMAPT* with CRISPR/Cas9-mediated homologous recombination. For each variant, 2 sgRNA sequences were selected flanking the variant site, as well as a donor homology template ssODN with at least 35bp of homology flanking each cut site. In the ssODN, DNA sequence was recoded to insert the desired variant along with silent mutations that block recutting by Cas9. The ssODN was co-injected into NMX329 with the appropriate sgRNAs and Cas9 and *dpy-10* co-CRISPR reagents. Animals were screened visually for possible transgene insertion using *dpy-10* . The desired edit was verified by PCR confirmed by DNA sequencing, and wild-type sequences were confirmed at the *dpy-10* co-CRISPR locus.

Statistical analysis: Student’s t-test used for comparison of neurodegeneration (Microsoft Excel). Kalpan Meier comparison used for survival to Day 9 (www.statskingdom.com/kaplan-meier.html)

## Reagents

**Table:** 

Gene	Outcrossed strain	Original strain	Genotype	
*ptl-1(-)*	HA3699	NMX41	*ptl-1(tgx30 [1729bp deletion]) III*	This study
*hMAPT(WT)*	HA3982	NMX329	*ptl-1(tgx315 [pNU2344-hMAPT WT ]) III*	This study
*hMAPT(G272V)*	HA3988	NMX372	*ptl-1(tgx315tgx354 [pNU2344-hMAPT G272V]) III*	This study
*hMAPT(G272V)*	HA3989	NMX373	*ptl-1(tgx315tgx355 [pNU2344-hMAPT G272V]) III*	This study
*hMAPT(P301L)*	HA4004	NMX405	*ptl-1(tgx315tgx381 [pNU2344-hMAPT P301L]) III*	This study
*alfa-1(-)*	HA3701	NMX62	*alfa-1(tgx53 [4052bp deletion]) II*	This study
*pgrn-1(-)*	HA3705	NMX90	*pgrn-1 (tgx81 [2004bp deletion]) I*	This study
*pha-1(ts)*	-	GE24	*pha-1(e2123) III*	CGC
*unc-18(knu409)*	-	COP1403	*unc-18(knu409[56bp insertion]) X*	InVivo Biosystems
DiI			1,1'-Dioctadecyl **-** 3,3,3',3'-Tetramethylindocarbocyanine Perchlorate	Molecular Probes
XhoI			XhoI	New England Biochemicals
Cas9			Cas9 protein with NLS	PNA Bio
Hygromycin B			Hygromycin B	Gold Biotechnology

**Table:** 

reference	sgRNA 1	sgRNA 2	ssODN sequence or plasmid name	Source
*ptl-1(-)*	TAGTCAGGGCTCTTTTCCGG	AACGCAACACACGTTGCCGG	GTTTTAGGAAACTCAGTATAGTCAGGGCTCTTTTCTAAATAAATAAACTCGAGCGGAGGCGGAAACGTTCAAATCGAAAACAGGAAGC	This study
*hMAPT(WT)*	TAGTCAGGGCTCTTTTCCGG	TGAAAGCATATTATTAAGCG	pNU2344 - P2A::HumanMAPT WT::hygR	This study
*hMAPT(G272V)*	AATCTCAAGCACCAACCAGG	TCCAACGTCCAATCCAAATG	AAGATCGGATCCACCGAGAATCTCAAGCACCAACCCGGAGTGGGTAAAGTACAGATAATAAATAAAAAACTAGACCTATCTAATGTTCAGTCTAAATGCGGATCCAAGGACAATATCAAGCATGTCCCAG	This study
*hMAPT(P301L)*	TTGGTGCTTGAGATTCTCGG	ATCAAGCATGTCCCAGGAGG	CTTAAAGAACGTCAAGTCCAAGATCGGATCCACCGAAAACTTGAAGCATCAGCCAGGTGGGGGTAAAGTCCAGATTATTAATAAAAAACTAGACCTATCTAATGTACAGTCAAAGTGTGGGTCTAAAGATAACATAAAACACGTTCTTGGAGGAGGATCTGTCCAAATCGTCTACAAGCCAGTCG	This study
*alfa-1(-)*	AAGTTTTTATTCATCACACG	GCAAACTGCTGTATTTACAC	TAAAAACAAAAGATTATAAAGTTTTTATTCATCACCTCGAGTTTATTTATTTACACAGGCAACAAACGAAAGAATTTTCCTGTAGGTT	This study
*pgrn-1(-)*	CAGCTTCTGACAATTTTCGC	CTGGAATGTGAAGGATGATG	tagcggcaatttctgaagactgtcggaagccggcgCCTCCCCAGAAGTCCTCCAGTCCTAAATAAATAAACTCGAGATGAGGATGAGGATCAGATTTAAttttacacgttt	This study
*unc-18(knu409)*	ggatgtcccaggcgaacggg	N/A	GAGTCAGAGATGCAATGAAGTTGATGGTGCCACTTTTGATTGACCCAGCCGTGCGGTGTGAAGACCGCCT	This study

**Table:** 

Plasmid	Genotype	Description
pNU2344	*P2A::hMAPT WT::hygR*	Donor homology arms for insertion into *ptl-1* , P2A self-cleaving peptide, *C. elegans* optimized *hMAPT* coding sequence including 3 synthetic introns, hygromycin resistance cassette
pNU792	*eft-3p::Cas9::tbb-2* 3’UTR	612bp *eft-3* promoter, 4303bp Cas9 including 3 synthetic introns, NLS and HA-tag, 252bp *tbb-2* 3’UTR
